# Fabrication and Experimental Validation of a Sensitive and Robust Tactile Sensing Array with a Micro-Structured Porous Dielectric Layer

**DOI:** 10.3390/mi13101724

**Published:** 2022-10-12

**Authors:** Shengjie Yao, Jianping Yu, Xiaoliang Jiang, Junfei Xu, Kun Lan, Zhehe Yao

**Affiliations:** 1 Key Laboratory of Air-Driven Equipment of Zhejiang Province, Quzhou University, Quzhou 324000, China; 2 College of Mechanical Engineering, Zhejiang University of Technology, Hangzhou 310023, China

**Keywords:** tactile sensor, porous dielectric layer, capacitive sensor, electronic skin

## Abstract

The development of pressure sensors of high sensitivity and stable robustness over a broad range is indispensable for the future progress of electronic skin applicable to the detection of normal and shear pressures of various dynamic human motions. Herein, we present a flexible capacitive tactile sensing array that incorporates a porous dielectric layer with micro-patterned structures on the surface to enable the sensitive detection of normal and shear pressures. The proposed sensing array showed great pressure-sensing performance in the experiments, with a broad sensing range from several kPa to 150 kPa of normal pressure and 20 kPa of shear pressure. Sensitivities of 0.54%/kPa at 10 kPa and below, 0.45%/kPa between 10 kPa and 80 kPa, and 0.12%/kPa at 80 kPa and above were achieved for normal pressures. Meanwhile, for shear pressures, sensitivities up to 1.14%/kPa and 1.08%/kPa in *x* and *y* directions, respectively, and below 10 kPa, 0.73%/kPa, and 0.75%/kPa under shear pressure over 10 kPa were also validated. The performance of the finger-attached sensing array was also demonstrated, demonstrating which was a potential electronic skin to use in all kinds of wearable devices, including prosthetic hands, surgical robots, and other pressure monitoring systems.

## 1. Introduction

Urgent needs for domestic robotics capable of essential human–machine interactive communication able to take part in everyday life to compensate for deformities have rapidly promoted the development of all kinds of flexible sensors equivalent to human skin. Nevertheless, some more convenient and robust tactile sensing with all kinds of contact stimuli detection capacities, including force, pressure, temperature, humidity, and vibration, is always required to further popularize domestic robotics becoming commercially available [1,2,3,4]. Force-sensing, especially normal and shear forces measurement in real time, is considered a fundamental capability for tactile sensing in most human–machine interactive cases, which can be easily implemented under piezo-resistive [5,6,7,8,9], capacitive [10,11,12,13,14,15,16], piezoelectric [17,18,19,20], and optical [21,22,23,24] sensing principles. Among them, capacitive tactile sensors have already been confirmed as a substitute with human skin sensing capability in sensitivity, response time, and durability [1,2,3,10,11,12,13,14,15,16].

Good flexibility, high sensitivity, wide measuring range, fast responsiveness, and stable robustness are significant requirements for practical applications of tactile sensing devices. For capacitive sensors, sensitivity can be determined by numerous factors, with dielectric permittivity being a chief one. Compared with commonly applied polydimethylsiloxane (PDMS) and other flexible composites, air has the lowest permittivity as the dielectric material and, thus, provides the highest sensitivity in cases with the same structure. Therefore, this concept has been widely applied in numerous research studies during the last decade, the proposed capacitive sensing element showed decent sensitivity since there was nothing but air between every two electrodes. Such capacitive sensing elements were usually combined in particular patterns to realize normal and shear forces measurement, and cross-shaped walls or similar constructions were implemented between the sensing elements to fully avoid possible coupling interferences [25,26,27]. However, using air as a dielectric layer would rapidly lead the tactile sensing element into a nonlinear measuring range since two electrodes may easily have contact even under small forces, and if applied forces still increase, the sensing capacitors can be saturated.

For achieving long measurement range and still maintaining high sensitivity, great efforts have been observed, and implementing a dielectric layer into micro-structured patterns on the surface is one effective attempt. Liang et al. [12] presented a flexible capacitive tactile sensor array embedded with a truncated PDMS pyramid array as a dielectric layer. The truncated pyramid array was easily deformed under tiny forces, leading to high sensitivity under a small force and large measurement range. Boutry et al. [13] also arranged pyramid microstructures along nature-inspired phyllotaxis spirals; an e-skin that mimicked the interlocked dermis–epidermis interface in human skin provided increased sensitivity and excellent cycling stability. Cho et al. [16] presented a flexible capacitive pressure sensor that incorporated micro-patterned pyramidal ionic gels to enable ultrasensitive pressure detection with a broad sensing range from a few pascals to 50 kPa. The only inadequacy of such construction was that the molds for micro-structures were usually fabricated through the photolithography process, which is time consuming and cost-ineffective.

Thereafter, some other reported approaches enhanced sensor sensitivity by forming porous PDMS rather than patterning microstructures on the surface of a dielectric layer [28,29,30,31]. The particle-template method is one convenient and simple strategy to fabricate a highly deformable PDMS dielectric layer with excellent reproducibility and repeatability. Tang et al. [28] fabricated a new capacitive pressure sensor based on a porous CCTO-PDMS membrane; a thin membrane of 40 μm thickness was made using the doctor blade method, which can be applied to very small scenes. Kim et al. [29] proposed a simple fabrication process of a highly sensitive capacitive pressure sensor using a porous dielectric layer with cone-shaped patterns, which were prepared using microwave irradiation of an emulsion consisting of a sacrificial solvent and a pre-cured PDMS solution—the cone-shaped patterns on the surface would also further enhance the sensitivity.

In this study, we present the fabrication and experimental validation of a sensitive and robust tactile sensing array based on a micro-structured porous PDMS dielectric layer. The porous structures were prepared using perfluorotributylamine (C12F27N, MACKLIN, CHINA) as the sacrificial solvent mixed with pre-cured PDMS for its non-conduciveness and thermal and chemical stability. A cylindrical pillar was patterned in the center of every unit sensing element as a substitution for cross-shaped walls enabling full decoupling of applied normal and shear forces. A sensing array was successfully fabricated and attached to human fingers, and evaluations of sensitivity, repeatability, and response time all validated the proposed tactile sensor to be a potential electronic skin.

## 2. Methods and Fabrication

### 2.1. Design and Method of Three-Axial Tactile Sensing

**Figure 1:**
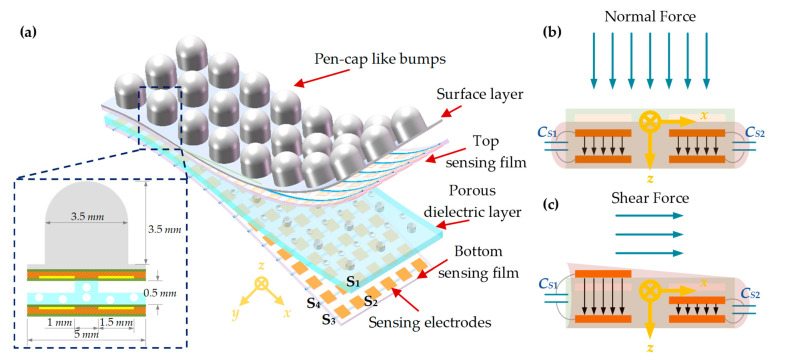
The schematic diagram of the capacitive tactile sensing array: (**a**) Detailed structure of unit sensing element; (**b**) A unit sensing element under normal force; (**c**) Under shear force.

The proposed electronic skin includes four layers: a polyimide (PI) sensing film with bottom electrodes, a micro-structured PDMS dielectric layer, a PI sensing film with top electrodes, and a surface layer with pen-cap-like bumps. The electronic skin is made of soft PI films and PDMS, which have been proven to have excellent temperature and chemical stability and mechanical durability. Every four top electrodes and confronted bottom electrodes consist of four sensing capacitors, namely S_1_, S_2_, S_3_, and S_4_, as shown in Figure 1a, making an individual unit sensing element.

A cylindrical pillar is patterned in the center of every unit sensing element and separates the top and bottom electrodes, leaving air between the narrow gaps to be the majority of the dielectric layer. Such a micro-structured surface of the dielectric layer enables every unit sensing element to be particularly sensitive to applied forces, the normal and shear components of which would also result in symmetrical impacts on four sensing capacitors, easily allowing for the full decoupling of multi-axial components in further calculations. As mentioned above, using air as the dielectric layer would rapidly lead to the saturation of the proposed sensor element, therefore, the PDMS consists of the dielectric layer fabricated into porous composition, which ensures the dielectric layer can be easily deformed under a small force but will not be completely compressed under a large force, thus, the sensor array can achieve high sensitivity under a small force as well as large measurement range.

The surface of the sensing array consists of numbers of pen-cap-like bumps for the reliable traction of objects. When normal or shear forces are applied on the surface, as shown in Figure 1b,c, the contacted bumps will be compressed or declined. The deflections of bumps will result in possible capacitance variations, which correspond to the applied normal and shear forces. Since the distance between the center points of two sensing electrodes in the diagonal direction is approximately 3.54 mm, bump diameter is set at 3.5 mm for reasonable density while maintaining adequate capacitance at each electrode. We fixed bump heights to 3.5 mm for sufficient shear load sensitivity; taller bumps increase the sensitivity while decrease the linearity.

### 2.2. Preparation of Micro-Structructured Porous PDMS Dielectric Layer

**Figure 2:**
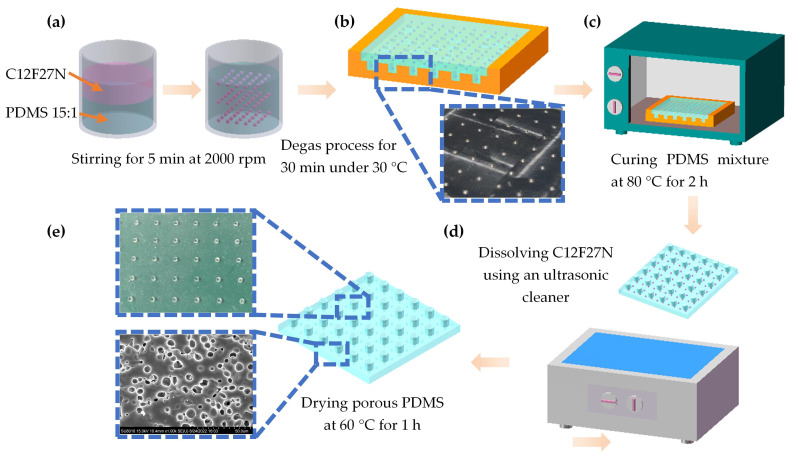
Schematic illustration of the fabrication process of porous PDMS dielectric layer: (**a**) Preparation of PDMS-C12F27N emulsion; (**b**) Molding and degassing process; (**c**) Curing of the PDMS mixture; (**d**) Dissolving C12F27N; (**e**) Dry process.

Figure 2 illustrates the fabrication process of a porous PDMS dielectric layer. C12F27N as the sacrificial solvent was used during the fabrication process for its non-conduciveness and thermal and chemical stability. Firstly, the PDMS was prepared by mixing a base gel and a curing agent (Sylgard 184, Dow Chemical Co., Milander, MI, USA) in a weight ratio of 15:1, as shown in Figure 2a, and C12F27N was dispersed in the pre-PDMS solution to fabricate the PDMS-C12F27N emulsion. Increasing the C12F27N ratio of the emulsion leads to a notable porous structure, thus, resulting in better sensitivity. However, we found that during the dispersion phase of C12F27N, it separated during the continuous phase when concentration was above a nearly 40% ratio in our tests, therefore, we expected that 30 vol.% of C12F27N would be a suitable concentration for a stable emulsion in the following preparation process. A stable emulsion was obtained under sufficient stirring for 5 min at 2000 rpm. The prepared mixture was poured on an aluminum mold with a dimension of 40 mm × 40 mm × 0.5 mm and placed in the vacuum desiccator to experience a further degas process for 30 min under 30 °C, as illustrated in Figure 2b. Possible residual air bubbles were evacuated in this step. Thereafter, as shown in Figure 2c,d, the mixture was cured on a hot-plate at 80 °C for 2 h, then the cured PDMS was washed for 6 h in deionized water (DI water) using an ultrasonic cleaner to dissolve the C12F27N. Finally, as shown in Figure 2e, the porous PDMS dielectric layer was dried at 60 °C for 1 h to remove the moisture. The photograph of the porous dielectric layer obtained by scanning electron microscope (HITACHI SU8010) illustrated that the pores were well-fabricated and of uniform size, as shown in Figure 2e.

### 2.3. Fabrication of Capacitive Tactile Sensor

**Figure 3:**
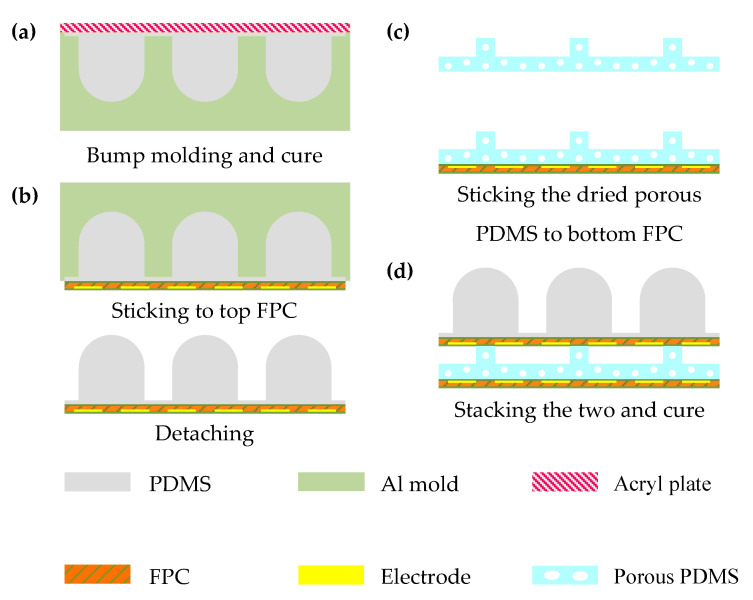
The specific fabrication process of tactile sensing array: (**a**) Bump molding and cure; (**b**) Sticking to top FPC and detaching; (**c**) Sticking the dried porous PDMS to bottom FPC; (**d**) Aligning and bonding all the layers of tactile sensing array.

The fabrication process of the proposed tactile sensor array is illustrated in Figure 3. Both the top and bottom electrodes were generated applying the FPC method. A double-sided board was selected in the design and electrodes and signal wires were fabricated on different layers to minimize the influences of parasitic capacitance. For the double-sided board, the base film was usually a 12.5 μm-thick PI film and two 18 μm-thick copper layers were bonded to separate sides of the base film by a 13 μm-thick adhesive layer, which made the thickness of the raw double-sided board 74.5 μm. By fabricating the sensing electrode using the FPC method, there is still the risk of possible micro-cracks, open welding, and other defects in unreasonable design or the manufacturing process but with a much lower possibility compared with being generated by the magnetron sputtering method. After drilling holes and plating through holes, electrodes and signal wires on two copper layers were electrically connected. Photoresist dry film was then pasted on the surfaces of the two copper layers and afterwards the designed electrode and signal wires patterns were transferred to the dry film under ultraviolet exposure, leaving the hollowed and transparent part of the desired copper retention area. After being washed by a certain developer, such as sodium carbonate, the uncured dry film was detached, fully exposing the undesired copper area. After developing, the exposed part of the copper layer was removed by etching solution, and the protected pattern was left. Remaining dry film was stripped by strong alkaline solution from the obtained board, making it suitable for the following solder mask printing, electroless nickel immersion gold (ENIG) method, and other finishing processes. Counting the thickness of the cover layers, the full thickness of the FPC board was eventually 110 μm. Another advantage of the FPC method along with the low risk of possible defects was that no extra transfer interface would be required, the signal wires on the FPC board can be connected to the following capacitance scanning circuit directly.

PDMS mixture was poured on an aluminum mold and spin-coated at 450 rpm for 30 s to fabricate the designed surface layer, as plotted in Figure 3a. The weight ratio of base gel and curing agent was 10:1, which resulted in a higher Young’s modulus than the dielectric layer. After a degas process for 20 min in the vacuum desiccator, remaining air bubbles were removed in this step. A smooth acrylic sheet was then laminated on the top of poured PDMS and the redundant PDMS was squeezed out by applying a light force on the acrylic sheet by hand. After curing the PDMS, the top PI sensing film was then firmly bonded to the bottom surface of the surface layer with silicone glue (Cemedine 8008) before peeling off from the PDMS mold, as shown in Figure 3b. Lastly, the dried porous dielectric layer and bottom PI sensing film were aligned and bonded to the top PI sensing film with the surface layer using a three-axial manual stage, as in Figure 3c,d, which ensured the precise and firm bonding of different layers of the proposed tactile sensing array.

**Figure 4:**
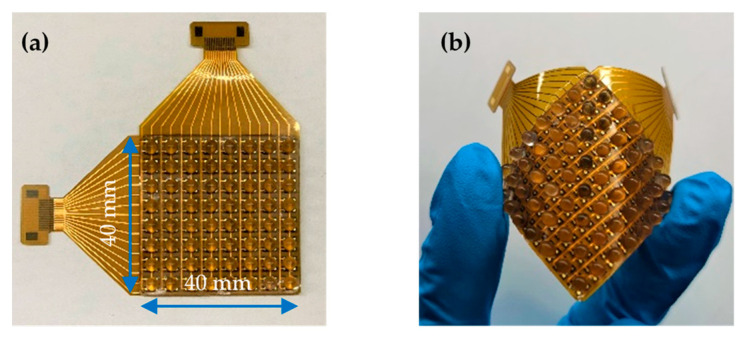
Illustration of the fabricated sensing array: (**a**) Placed on flat surface; (**b**) Bent by hand.

Figure 4a shows the fabricated sensor array, which was composed of 8 × 8 unit sensing elements with overall dimensions of 40 mm × 40 mm. The distance between two central points of the adjacent units showed that the spatial resolution was 5 mm. The fabricated sensor array featured high flexibility and was easily bent by hand, as shown in Figure 4b. 

## 3. Results and Discussion

### 3.1. Experimental Setup

**Figure 5:**
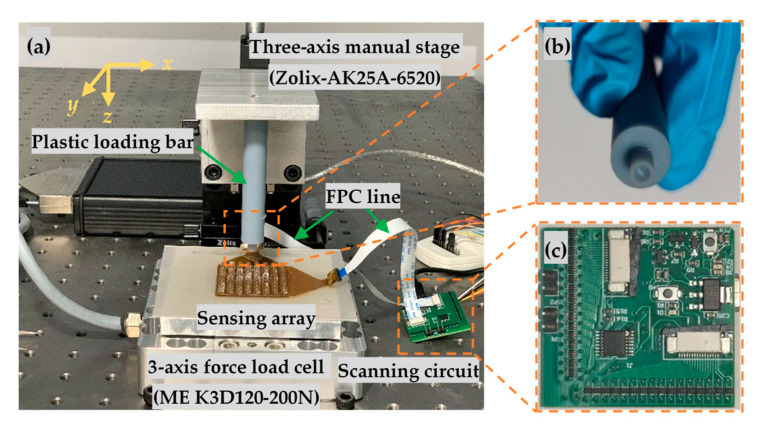
Experimental setup to study the sensing performance of the sensor array: (**a**) Overall view of the proposed test bench; (**b**) Plastic loading bar; (**c**) Scanning circuits for capacitance real-time measurement.

Figure 5a illustrates the basic composition of the test bench, in which the sensing array was firstly fixed to a glass wafer and then mounted on a three-dimensional force sensor (ME K3D120) with resolution up to 0.01 mN and relative linearity error down to 0.2%FS. A 3D printed (Nova, bena5) loading bar using white standard resin was mounted on a three-axial manual stage (Zolix-AK25A-6520), the front tip of which was designed as a small cylinder with a diameter of 4 mm. To simultaneously apply normal and shear forces on the bumps, the bottom surface of the front tip was fabricated as a hemisphere concave with the same shape as the bump, as shown in Figure 5b. In this case, the loading bar would always make full contact with the top surface of the bump of the unit sensing element. Along with the movement of the stage in the *x*, *y*, and *z* directions, the tip applies normal and shear force to the unit sensing element simultaneously.

The proposed tactile sensing array was designed with 8 × 8 common unit sensing elements, that detected 256 capacitors in a single measuring sequence, which was usually conducted based on scanning detection of a common setup, as shown in Figure 5c. The fabricated tactile sensing array in Figure 4 was directly connected to the scanning circuit via a commoditized 16-pin FPC connector (0.5K-AS-16PWB). During a full measurement sequence, all the sensing capacitors should be scanned in sequence, and for every clock cycle, only one analog switch in a row and column would be connected, thus, one specified capacitor was selected, while the rest of the capacitors were shielded by the virtual ground. Capacitance values were measured by a capacitance-to-digital converter (AD7745), whose update rate ranged from 10 Hz to 90 Hz. In this experiment, a sampling rate of AD7745 was set to 50 Hz, achieving higher measuring efficiency.

### 3.2. Sensing Array Calibration

**Figure 6:**
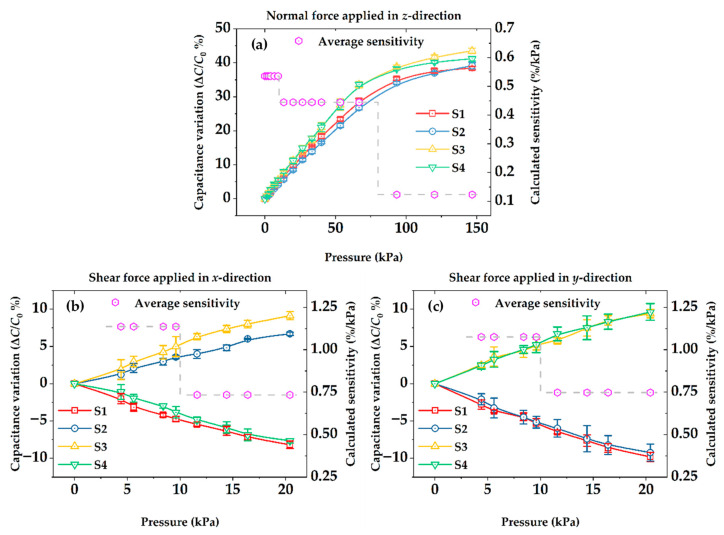
Illustration of the pressure-sensing properties of a unit sensing element to external forces: (**a**) Normal force applied in *z* direction; (**b**) Shear force applied in *x* direction; (**c**) Shear force applied in *y* direction.

Sensitivity, commonly defined as *S* = (Δ*C*/*C*_0_)/*p* × 100%, was calibrated firstly based on the proposed test bench, where *p* represents the applied normal or shear pressures, while Δ*C* and *C*_0_ are the capacitance variations of the measured sensor and the initial capacitance value without external forces, respectively. Considering that all unit sensing elements of the sensor array shared the same pattern, the unit sensing element in row 4 and column 4 was selected as the specific calibration object. Capacitance, known as *ε*_0_*ε_r_A_s_*/*g,* would not be in a close linear relationship to applied external forces, where *ε_r_* denotes the dielectric constant of porous PDMS and *ε*_0_ denotes that of pure air, while *A_s_* and *g* represent the sensing area and gap distance, respectively; thereafter, plotted in Figure 6a, average sensitivity between three different intervals ranging from 1 kPa to 150 kPa in the *z* direction were calculated to evaluate sensing array performance of applying normal forces. The results imply that sensitivity as a function of applied pressure decreased along with the increase in external pressures, sensitivity up to 0.54%/kPa was obtained when the pressure was below 10 kPa, decreasing to 0.12%/kPa at a pressure of 80 kPa and above. 

Shear forces were relatively lower compared with applying normal forces in most cases; therefore, as shown in Figure 6b,c, average sensitivities between every 10 kPa, ranging from 0 to 20 kPa in the *x* and *y* directions, were calculated to evaluate sensing array performance of applying shear forces, respectively. Due to the pen-cap-like bumps on the surface of sensing array, sensitivities of shear forces up to 1.14%/kPa and 1.08%/kPa were obtained in the *x* and *y* directions, respectively, when the pressure was below 10 kPa, and slightly decreased to 0.73%/kPa and 0.75%/kPa at shear pressures over 10 kPa, whose performances were considered better than that of normal forces. 

Compared with the existing three-axial capacitive tactile sensor using PDMS truncated pyramids, a dielectric layer featured sensitivities of 0.93 and 0.92%/kPa in *x* and *y* directions at shear pressure below 31.25 kPa, meanwhile it was 1.08%/kPa for normal pressure below 31.25 kPa and 0.123%/kPa between 31.25 kPa and 250 kPa [12]. The developed sensing array provided higher sensitivity only for shear pressures, which we assumed acceptable at the present stage considering the entire fabrication process was simple and of low expense. Another reported capacitive sensor applied in the electronic skin system using a porous dielectric layer featured sensitivity of 2.3%/kPa for normal pressure only below 20 kPa [30], but the thickness of the dielectric layer was 4.5 mm in the presented tests, which was nine time thicker than our proposed sensing array. Overall speaking, the capability of providing good sensitivity for gentle touch or soft contact force and large measuring range at the same time based on the proposed tactile sensing array have been demonstrated during the experiments, which was suitable for possible robotics and prosthetic hand applications.

**Figure 7:**
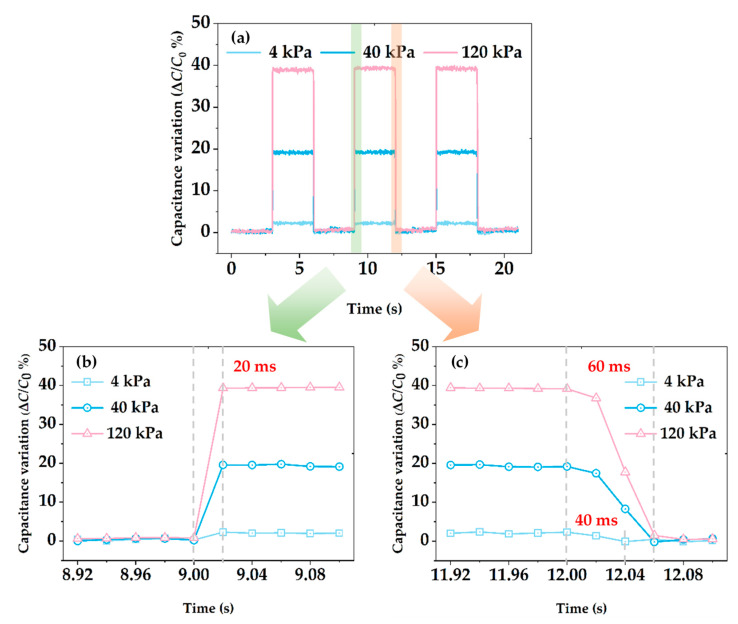
Time-resolved capacitance change response of the sensor: (**a**) Under repeated external loads with different pressures ranging from 4 to 120 kPa; (**b**) Response time measured during repeated loading; (**c**) Response time measured during repeated unloading.

In addition, fast responses were observed over the broad range of applied normal pressure (4 kPa, 40 kPa, and 120 kPa) in a stepwise manner by repeating loading for 3 s and unloading for 3 s, as shown in Figure 7a. Stable output signals have been acquired during three times of repeated cycles, confirming that the proposed sensing array was capable of operating under a diverse pressure regime with sufficient repeatability, which was mainly attributed to the excellent elastic property of the porous structure and full restoration of PDMS walls after buckling under compressive pressure. During repeated loading, the unit sensing element can always react with the minimum measurement interval—a response time of less than 20 ms was measured, as shown in Figure 7b.

However, during repeated unloading, as plotted in Figure 7c, the recovery time of the unit sensing element was gradually extended from 40 to 60 ms as the loading pressure increased, which was attributed to hysteresis characteristic of the dielectric layer. Therefore, it can be concluded that the unit sensing element responded quickly to the external pressure and can better recover to the initial capacitance value after the external pressure was unloaded. For the entire tactile sensing array, capacitances of each unit sensing element were measured in sequence, a complete measuring cycle lasted for 5.12 s, and slight recovery time delay played negligible impacts on identifying the overall pressure distribution in real-time. Nevertheless, different efforts still need to be attempted to improve the dynamic response speed as well as the measuring speed of the scanning circuit for faster response times of the tactile sensing array.

### 3.3. Three-Axial Force Sensing

The capacitance variations of the proposed tactile sensing array are mainly determined by gap distance changes, which are closely related to applied forces. When a normal force is applied on the bumps of the surface layer, the contacted bumps will be compressed in the *z* direction, gap distances of four sensing capacitors attain the same variation, defined as *g_z_*. When a shear force in the *x* direction is loaded, the contacted bumps will be declined to the same direction, the gap distances of *S*_2_ and *S*_3_ decrease with the same variation as *g_x_*, while those of *S*_1_ and *S*_4_ increase to the same value. When a shear force in the *y* direction is loaded, the gap distances of *S*_1_ and *S*_2_ increase with the same variation as *g_y_*, while those of *S*_3_ and *S*_4_ decrease. Once gap variations induced by specific forces in diverse directions are clarified, the applied external force can easily be divided into its normal force component *F_z_* and shear force components *F_x_* and *F_y_* simultaneously. In this concern, the relationships between capacitance variations with gap distances changes are established as the following:

(1) $$\begin{array}{l} 1/C_{S1}-1/C_{S10}=\Delta g_{1}/\varepsilon_{0}\varepsilon_{r}A_{S}=\left(-g_{z}+g_{x}+g_{y}\right)/\varepsilon_{0}\varepsilon_{r}A_{S}\\ 1/C_{S2}-1/C_{S20}=\Delta g_{2}/\varepsilon_{0}\varepsilon_{r}A_{S}=\left(-g_{z}-g_{x}+g_{y}\right)/\varepsilon_{0}\varepsilon_{r}A_{S}\\ 1/C_{S3}-1/C_{S30}=\Delta g_{3}/\varepsilon_{0}\varepsilon_{r}A_{S}=\left(-g_{z}-g_{x}-g_{y}\right)/\varepsilon_{0}\varepsilon_{r}A_{S}\\ 1/C_{S4}-1/C_{S40}=\Delta g_{4}/\varepsilon_{0}\varepsilon_{r}A_{S}=\left(-g_{z}+g_{x}-g_{y}\right)/\varepsilon_{0}\varepsilon_{r}A_{S} \end{array}$$

where Δ*g_i_* denotes the gap distance change of each capacitor in a unit sensing element, *C_Si_*_0_ and *C_Si_* are the initial and current capacitances of the unit sensing element, respectively.

When capacitance variations of every unit sensing element are detected, gap distance changes in four sensing capacitors can easily be calculated from Equation (1), then gap variations *g_m_* (*m* = *x*, *y*, *z*) derived by normal and shear forces *F_m_* (*m* = *x*, *y*, *z*) can be achieved as the following:

(2) $$\begin{array}{l} g_{x}=1/4\left(\Delta g_{1}-\Delta g_{2}-\Delta g_{3}+\Delta g_{4}\right)\\ g_{y}=1/4\left(\Delta g_{1}+\Delta g_{2}-\Delta g_{3}-\Delta g_{4}\right)\\ g_{z}=-1/4\left(\Delta g_{1}+\Delta g_{2}+\Delta g_{3}+\Delta g_{4}\right) \end{array}$$

In this study, the relationships between applied forces and gap variation should be defined based on acquired data from former sensing array calibration due to the nonlinearity between the forces and capacitance changes, as shown in Figure 6. Therefore, specific relations between *F_m_* and *g_m_* are claimed as follows after polynomial fitting:

(3) $$\begin{array}{l} F_{x}=1.49g_{x}-3.00g_{x}{}^{2}+6.21g_{x}{}^{3}\\ F_{y}=0.96g_{y}-0.70g_{y}{}^{2}+1.88g_{y}{}^{3}\\ F_{z}=-0.28g_{z}+10.68g_{z}{}^{2}-16.12g_{z}{}^{3}+7.67g_{z}{}^{4} \end{array}$$

R-square of fitted curves of *F_x_*, *F_y_*, and *F_z_* were 0.998, 0.999, and 0.995, respectively, which implies that three-axial external forces can be precisely detected when provided with accurately measured capacitance variations.

### 3.4. Distributed Contact Force Sensing

**Figure 8:**
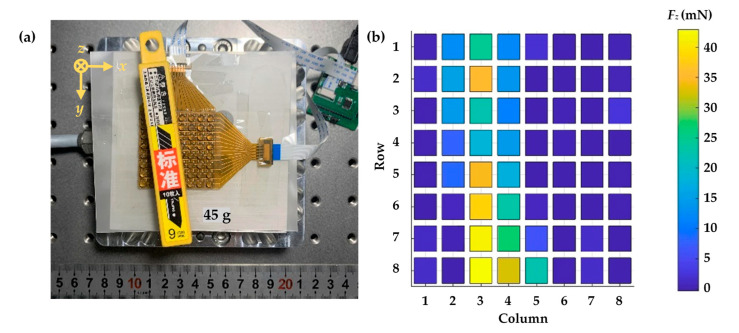
Normal pressure distribution test when a blade box (45 g) rested on the sensing array: (**a**) Overview of experimental setup; (**b**) Real-time overall pressure distribution.

Accurately detecting the pressure distribution is a critical factor for measuring fine movements as an electronic skin. Compared to a single unit sensing element, the full sensing array is arranged as a matrix, similar to biological skin and, thus, can more effectively identify the overall pressure distribution. Based on the proposed scanning circuits, three-axial force components (*F_ax_*, *F_y_*, and *F_z_*) measured by the full sensing array are transmitted to the host computer and displayed in real-time. For demonstration, a plastic box of flat surfaces filled with blades weighing 45 g was placed on the sensing array as shown ni Figure 8a, the weight of which was distributed to contact areas. Measuring results in Figure 8b demonstrate that the sensing array could accurately map the distribution of the capacitance responses corresponding to the shape and weight of the objects, and that would be critical in a real grasping situation. 

**Figure 9:**
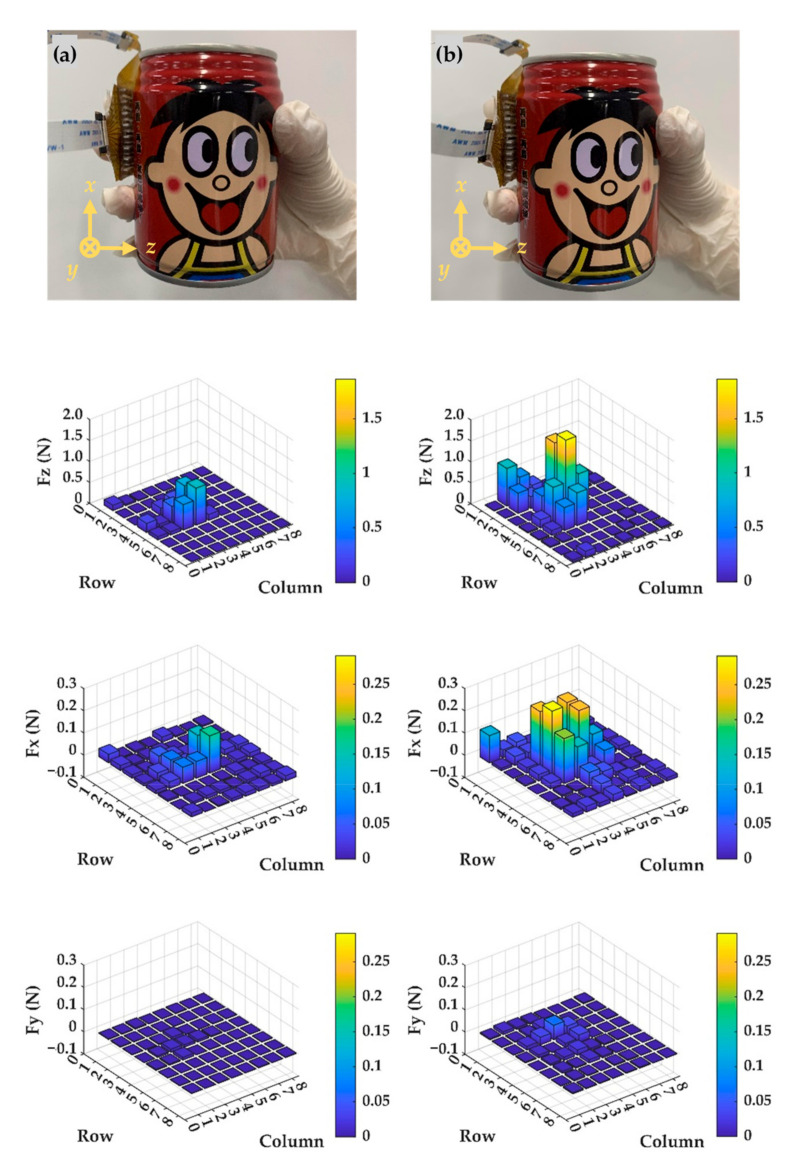
The measured contact force distribution with the sensing array mounted on two fingers: (**a**) When picking up an empty beverage can; (**b**) Grasping a full one.

Furthermore, we also demonstrated the measurements of dynamic pressures to apply the tactile sensing array as an electronic skin. The proposed tactile sensing array was firstly attached to one author’s hands with two fingers using double-sided tape, then picking up an empty beverage can and a full one under the same measuring circumstances, as shown in Figure 9a,b, respectively. Specific real-time distribution of three-axial force components among the full measurement regime of the proposed sensing array was well-illustrated. Each unit sensing element responded well to the dynamic motions and maintenance of, and the increase and decrease in, applied external forces in terms of capacitance, variations were all well-detected in real-time. For the empty beverage can, the weight can be neglected, thus, picking up and releasing caused minimal differences in detected shear force components *F_x_* and *F_y_*. However, when the can was full, higher grasping force was required for stead holding. Therefore, compared with picking up an empty one, larger normal force component *F_z_* was detected, meanwhile, as weight of the full can increased, a positive value of *F_x_* was detected, as the results in Figure 9b show. These demonstrations indicate that the proposed tactile sensing array is a qualified electronic skin that can be applied to all kinds of medical instruments that require real-time pressure monitoring.

## 4. Conclusions

In summary, we demonstrated that a porous dielectric layer, fabricated from self-made PDMS-C12F27N emulsion, was useful for sensing normal and shear pressures over a broad range up to 150 kPa of normal pressure and 20 kPa of shear pressure with high sensitivities. The proposed capacitive tactile sensing array exhibited sensitivities of 0.54%/kPa at 10 kPa and below, 0.45%/kPa between 10 kPa and 80 kPa, and 0.12%/kPa at 80 kPa and above of normal pressures. Meanwhile, for shear pressures, our sensing array displayed sensitivities up to 1.14%/kPa and 1.08%/kPa in X and Y directions, respectively, at 10 kPa and below, and slightly decreased to 0.73%/kPa and 0.75%/kPa when shear pressure was over 10 kPa. The fast capacitance response to applied normal pressure and sufficient repeatability over multiple pressure cycles were attributed to the excellent elastic property of the porous dielectric layer that resulted from its higher air-PDMS ratio and full restoration of PDMS walls. Furthermore, distributed contact force sensing tests validated that our robust, highly sensitive, and broad pressure range tactile sensing array was suitable for the efficient detection of a variety of pressure sources under different circumstances, including, but not limited to, finger touching, hand grasping, human impulses, breathing, and other gentle human motions, which merits further study.

## Data Availability

Not applicable.
